# Risk factors predicting graft-versus-host disease and relapse-free survival after allogeneic hematopoietic stem cell transplantation in relapsed or refractory non-Hodgkin’s lymphoma

**DOI:** 10.1007/s00277-019-03714-x

**Published:** 2019-05-14

**Authors:** Young-Woo Jeon, Seugyun Yoon, Gi June Min, Sung-Soo Park, Silvia Park, Jae-Ho Yoon, Sung-Eun Lee, Byung-Sik Cho, Ki-Seong Eom, Yoo-Jin Kim, Hee-Je Kim, Seok Lee, Chang-Ki Min, Jong Wook Lee, Seok-Goo Cho

**Affiliations:** 1 Division of Lymphoma-Myeloma, Catholic Hematology Hospital, Seoul St. Mary’s Hospital, College of Medicine, The Catholic University of Korea, #505, Banpo-Dong, Seocho-Ku, Seoul, 06591 Republic of Korea; 2 Leukemia Research Institute, College of Medicine, The Catholic University of Korea, Seoul, Republic of Korea; 3 Institute for Translational Research and Molecular Imaging, Catholic Institutes of Medical Science, Seoul, Republic of Korea; 4 Laboratory of Immune Regulation, Convergent Research Consortium for Immunologic Disease, Seoul St. Mary’s Hospital, Seoul, Republic of Korea

**Keywords:** Allogeneic hematopoietic stem cell transplantation, Endpoint, Graft-versus-host disease, Lymphoma

## Abstract

Allogeneic hematopoietic stem cell transplantation (allo-HSCT) is still considered a definitive curative modality for refractory or relapsed non-Hodgkin’s lymphoma (NHL). However, transplant-related morbidity and mortality remain a considerable challenge. The graft-versus-host disease (GVHD)–free with relapse-free survival (GRFS) rate and GRFS-related prognostic factors have not been fully examined for NHL alone. We evaluated 104 consecutive patients with refractory or relapsed aggressive NHL receiving allo-HSCT at a single institution. With a median follow-up of 31.5 months, the estimated 3-year overall survival (OS), disease-free survival (DFS), the cumulative incidence rates of relapse, and non-relapse mortality were 45.9%, 45.9%, 36.0%, and 17.0%, respectively. The patients with overall grades III–IV acute GVHD had markedly inferior OS and DFS (*p* = 0.040 for OS and *p* = 0.028 for DFS). However, patients with more than mild stage chronic GVHD showed superior OS and DFS (*p* = 0.004 and *p* = 0.008, respectively). The 1- and 3-year GRFS rates were 44.5% and 36.9%, respectively. The negative bone marrow involvement at diagnosis, chemosensitive disease status, and fewer exposure lines of chemotherapy before transplantation significantly increased the GRFS incidence. However, no transplant-associated factors were related to GRFS incidence. Furthermore, applying dynamic GRFS method which excepted patients whose chronic GVHD was fully resolved within short-period, survival rate significantly increased over time (36.9% vs. 41.9%, *p* = 0.045 for conventional GRFS vs. dynamic GRFS at 3 years after transplantation). In conclusion, these results suggest that GRFS is also a useful endpoint to assess transplant outcomes, and the dynamic GRFS calculation, including rapidly manageable chronic GVHD, is a more practical method for patients with refractory or relapsed heterogenous subtypes of NHL.

**Electronic supplementary material:**

The online version of this article (10.1007/s00277-019-03714-x) contains supplementary material, which is available to authorized users.

## Introduction

Since rituximab-based chemoimmunotherapy and several novel targeted therapies were introduced, the survival outcomes of patients on the spectrum of non-Hodgkin’s lymphoma (NHL), from indolent to aggressive subtypes, have improved drastically [1, 2]. However, approximately half of patients with advanced NHL fail to achieve a complete response (CR), and numerous salvage treatments with concomitant novel agents or autologous hematopoietic stem cell transplantation (auto-HSCT) have been applied to overcome these obstacles depending on the patient’s clinicopathological condition. However, final survival outcomes have been disappointing [3–5].

In this circumstance, allogeneic hematopoietic stem cell transplantation (allo-HSCT) is considered a potentially curative option in patients experiencing relapse after auto-HSCT, and in those who are chemorefractory after multiple chemotherapy regimens [6]. As allo-HSCT procedures have evolved over the years according to changes in conditioning intensity and alternative donor selection, 5-year overall survival (OS) and progression-free survival (PFS) rates have reached 30–65% and 37–60%, respectively [6–8]. Survival outcome was thought to be predicated on the use of tumor-free grafts, as well as a potential allogeneic effect exerted by donor T cells, known as the graft-versus-lymphoma (GVL) effect. However, adverse events, such as transplant-related complications and disease recurrence, are the most troublesome [9]; acute graft-versus-host disease (GVHD) or chronic GVHD is related to poor non-relapse mortality (NRM) and decreased the quality of life (QoL). Disease recurrence after allo-HSCT and transplant-related mortality (TRM) have failed to maintain satisfactory allo-HSCT OS and DFS rates [9].

Most clinical studies on allo-HSCT have focused primarily on OS or DFS and have only evaluated the effectiveness of the transplantation itself. However, a major drawback in this kind of analysis is that these studies dealt separately with transplant-related complications, which significantly decrease QoL, such as severe acute GVHD and extensive chronic GVHD, and may not reflect the ultimate efficacy of allo-HSCT. Thus, there is a need to appropriately evaluate the objective effectiveness of allo-HSCT and the subjective improvement in the patient’s experience. To compensate for this pitfall, Holtan et al. proposed that the novel composite endpoint of GVHD-free relapse-free survival (GRFS) after HSCT be defined as survival without grades III–IV acute GVHD, moderate to severe chronic GVHD requiring systemic immunosuppressive therapy, relapse, or death [10, 11]. GRFS is now widely used to measure the success of HSCT in many hematological malignancies, such as acute leukemia and myelodysplastic syndrome, but research on NHL-specific GRFS is relatively limited because NHL is not a disease considered to be upfront for allo-HSCT at the initial diagnosis. Thus, candidates with NHL for allo-HSCT are inevitably exposed to multiple lines of chemotherapy in a chemosensitive or chemorefractory relapse pattern, in addition to a reduced intensity conditioning (RIC) regimen.

Therefore, it is essential to understand the equivalent survival outcomes between post-HSCT disease relapse and transplant-related GVHD for relapsed or refractory patients with NHL. We identified the GRFS rate as an effective modality influencing practical survival outcomes in patients with NHL alone and examined the GRFS-associated prognostic factors for patients with far-advanced NHL alone.

## Materials and methods

### Study design

Adult patients who underwent allo-HSCT for relapsed or refractory lymphoma from January 2008 to January 2017 at our institute were screened, and subjects who were diagnosed pathologically with NHL were selected for the analysis. The observations were extended until April 2018 to ensure a minimum follow-up duration of 12 months. Clinical data were retrospectively extracted from the patient’s electronic medical records, including demographic information, initial or salvage chemotherapy, response to initial or salvage chemotherapy, high dose chemotherapy followed by autologous stem cell rescue, disease status at allo-HSCT, and outcome. We treated 104 relapsed or refractory consecutive patients with RIC regimens or myeloablative conditioning (MAC) regimens after several salvage chemotherapies. Experienced lymphoma pathologists confirmed the pathology according to the 2008 World Health Organization classification [12].

### Human leukocyte antigen typing

Human leukocyte antigen (HLA) typing for HLA-A, HLA-B, HAL-C, and HLA-DR was performed on all patients and donors to ensure appropriate matches; “well matched” was defined as non-disparity between the donor and recipient at HLA-A, B, C, and DR1 (8/8), “partially matched” was a single known or likely disparity, “mismatched” was two or more disparities, and haploidentical donors were those with a 6-4/8 HLA matching degree [13]. Sibling or unrelated donors were classified according to donor type.

### Conditioning regimen and prophylaxis for graft-versus-host disease

The MAC regimen consisted of cyclophosphamide (120 mg/kg) and 30 mg/kg etoposide in combination with total body irradiation (TBI; 1200 cGy in four fractionated doses over 4 days). RIC mainly included 30 mg/m^2^ fludarabine for 6 consecutive days (total, 180 mg/m^2^) plus 70 mg/m^2^ melphalan for 1 day with additional TBI of 800 cGy in four fractionated doses for 2 days (FMT regimen). Another RIC regimen was 30 mg/m^2^ fludarabine for 6 consecutive days (total, 180 mg/m^2^) plus 1.6 mg/kg busulfan for 2 days (total, 3.2 mg/kg) with 800 cGy TBI for 2 days. Anti-thymocyte globulin (rabbit ATG, 2.5–5.0 mg/kg; Genzyme Transplant, Cambridge, MA, USA) was administered as part of the conditioning regimen for some patients treated with RIC, 1.25 mg/kg for 2 days (total, 2.5 mg/kg) in patients receiving from one allele mismatched to one antigen mismatched donor, and 2.5 mg/kg for 2 days in patients with a haploidentical donor regardless of whether it was a sibling or unrelated. Moreover, GVHD prophylaxis mainly consisted of a calcineurin inhibitor (cyclosporine for all sibling transplants and tacrolimus for unrelated transplants or haploidentical transplants) with a short course of methotrexate (5 mg/m^2^ for tacrolimus and 10 mg/m^2^ for cyclosporine) on days + 1, + 3, + 6, and + 11 during the transplant period. There was no post-transplant cyclophosphamide (PT-CY)-based GVHD prophylaxis strategy in our cohort. The MAC regimen was selected for younger patients with good general health status in addition to CR disease status before strict allo-HSCT. Patients who did not meet the conditions for MAC were treated with RIC, and haploidentical transplants were adopted with the same RIC method. All patients were managed in a specific sterilized room with laminar airflow and high-efficacy air purification filters. Acyclovir and itraconazole were prescribed to all patients for viral and fungal prophylaxis. All patients received granulocyte-colony stimulating factor (filgrastim) beginning on the day when the absolute neutrophil count (ANC) was < 0.5 × 10^9^ cells/L at a dose of 5 μg/kg/day subcutaneously until the ANC was > 1.0 × 10^9^ cells/L. Other conservative management was performed according to the event.

### Clinical survival outcomes and evaluation of transplant-related risks

The primary outcomes were 1- and 3-year GRFS for patients with NHL alone, and current GRFS was evaluated as the composite in the absence of grades III–IV acute GVHD, systemic immunosuppressive therapy–requiring chronic GVHD, relapse, or death from any cause, during each time point after allo-HSCT [10]. The prognostic parameters for GRFS were assessed at 1 and 3 years after allo-HSCT. If multiple GRFS-related events occurred in one patient, the first post-transplant event was recognized within 1 and 3 years. Secondary outcomes evaluated OS and PFS, the cumulative incidence of GRFS-related acute or chronic GVHD, relapse, and NRM. Additionally, transplant-related risk was calculated by the European Group for Blood and Marrow Transplantation (EBMT) scoring system, including age at diagnosis and HSCT, pre-HSCT status, and donor-recipient combinations [14]. Chemotherapy-related toxicity was calculated using the National Cancer Institute Common Toxicity Criteria for Adverse Events (ver. 4.0), and acute GVHD and chronic GVHD were diagnosed and graded according to the system of Glucksberg/Thomas and the National Institutes of Health Consensus [15, 16].

### Statistical analysis

Surviving patients were censored on the last day of follow-up. All GRFS-associated categorical variables are expressed as proportions and compared with the chi-square or Fisher’s exact test, and continuous variables are presented as median with range and compared using the Mann–Whitney *U* test between the two groups. OS, DFS, and GRFS rates were calculated using the Kaplan–Meier survival method in a log-rank analysis. Cumulative incidence estimates of acute GVHD, chronic GVHD, relapse, and NRM were calculated with relapse or death from other causes defined as competitive events, using the Gray test for univariate analysis and the Fine–Gray method for the proportional hazard regression. All statistical analyses were performed using R software (ver. 3.2.0; Comprehensive R Archive Network project, http://cran.us.r-project.org) with the EZR graphical user interface of Y. Kanda (Saitama Medical Center, Jichi Medical University, Saitama, Japan) [17].

## Results

### Patient characteristics

The baseline clinical characteristics of the 104 patients included in the study are summarized in Table 1. The median ages at the initial diagnosis and allo-HSCT of the entire cohort were 39 (range 18–64 years) and 40 years (range 19–65 years), respectively. Moreover, the proportion of male patients was higher (*n* = 68, 65.4%). The pathological phenotype distribution was 50 patients (48.1%) with B cell NHL and 54 patients (51.9%) with T cell NHL, and specific subtypes of B cell and T cell NHL were also shown in Table 1. The majority of patients presented with advanced disease status at the initial diagnosis, Ann Arbor stage IV (*n* = 59, 56.7%), elevated serum lactate dehydrogenase (*n* = 67, 64.4%), involvement of two or more lymph nodes (*n* = 67, 64.4%), and bone marrow (BM) involvement (*n* = 46, 44.2%). Eighty-eight patients (84.6%) had a relatively favorable Eastern Oncology Group performance score of 0–1. The mean number of systemic chemotherapy regimens before allo-HSCT was four. Moreover, 11 (10.6%) patients were treated with five or more rounds of chemotherapy, and 38 patients (36.5%) developed progressive disease after an autologous stem cell transplant. Accordingly, the interval from the initial diagnosis to transplant was > 12 months (*n* = 72, 69.2%). Only 35 patients (33.7%) had CR status before allo-HSCT, and 26 patients (25%) had PR; in other words, fewer than half of all patients were in a relapsed or refractory state after final salvage chemotherapy pre-HSCT (43 patients with SD or PD, 41.3%).

**Table 1:** Patient characteristics

Factors	*N* = 104 (%)
Age, year, median (range) at initial diagnosis	39 (18–64)
Gender, male (%)	68 (65.4)
Pathological subtype (%)	
Diffuse large B cell lymphoma	30 (28.8)
T cell lymphoblastic lymphoma	17 (16.4)
Peripheral T cell lymphoma, NOS	13 (12.5)
Extranodal NK/T cell lymphoma-nasal type	9 (8.7)
B cell lymphoblastic lymphoma	8 (7.7)
Mantle cell lymphoma	6 (5.8)
Angioimmunoblastic T cell lymphoma	6 (5.8)
Aggressive NK cell lymphoma	6 (5.8)
Follicular lymphoma	2 (1.9)
Plasmablastic lymphoma	2 (1.9)
Others*	5 (4.7)
International Prognostic Index (IPI) at initial diagnosis	
Low	33 (31.7)
Low-intermediate	32 (30.8)
High-intermediate	27 (26.0)
High	12 (11.5)
Ann Arbor stage at initial diagnosis	
I	2 (1.9)
II	25 (24.0)
III	18 (17.3)
IV	59 (56.7)
LDH at initial diagnosis	
Normal	44 (42.3)
Elevated (> 450 IU/L)	60 (57.7)
Extranodal lymph node involvement (≥ 2)	67 (64.4)
ECOG PS at initial diagnosis	
0–1	88 (84.6)
≥ 2	16 (15.4)
Bone marrow involvement at initial diagnosis	46 (44.2)
Bone marrow involvement before allo-HSCT	13 (12.5%)
Beta2-microglobulin	
Normal	44 (42.3)
Elevated (≥ 2.5 mg/L)	37 (35.6)
Not assessed	23 (22.1)
History of prior auto-HSCT	38 (36.5)
no	66 (63.5)
yes	38 (36.5)
Lines of chemotherapy before allo-HSCT	
1	1 (1.0)
2	24 (23.0)
3	26 (25)
4	42 (40.4)
≥ 5	11 (10.6)
Disease status at allo-HSCT	
CR	35 (33.7)
PR	26 (25)
SD/PD	43 (41.3)
Interval period from diagnosis to transplant	
< 12 months	32 (30.8)
12–24 months	28 (26.9)
>24 months	44 (42.3)

*Others: anaplastic large cell lymphoma, chronic lymphocytic leukemia, enteropathy-associated T cell lymphoma, hepatosplenic T cell lymphoma, and subcutaneous panniculitis T cell lymphoma

*NOS*, not otherwise specified; *NK*, natural killer; *LDH*, lactate dehydrogenase; *ECOG*, Eastern Cooperative Oncology Group performance status; *HSCT*, hematopoietic stem cell transplantation; *CR*, complete response; *PR*, partial response; *SD*, stable disease; *PD*, progressive disease

### Transplantation-related characteristics

The transplant-associated characteristics are given in Table 2. Peripheral blood was the source of stem cells for most patients (*n* = 99, 95.3%). The majority of patients were treated with the FMT conditioning regimen: 76 patients (73.1%) received the FMT conditioning regimen, 19 patients (18.3%) received the fludarabine–busulfan regimen, and 9 (8.6%) were transplanted with the MAC regimen. Fewer than half of all patients (*n* = 42, 40.4%) were treated with ATG as part of their conditioning regimen. Donors for 34 patients (32.7%) were HLA-identical siblings, 35 patients (33.2%) were transplanted with HLA-identical unrelated, and a considerable number of patients (*n* = 22, 21.6%) were infused with HLA-haploidentical stem cells.

**Table 2:** Allogeneic stem cell transplantation-related characteristics

Factors	*N* = 104 (%)
HCT-CI (score)	
0	35 (33.7)
1–2	41 (39.4)
≥ 3	28 (26.9)
Conditioning regimen	
RIC 1 (Flu + Mel + TBI)	76 (73.1)
RIC 2 (Flu + Bu)	19 (18.3)
MAC (Cy + Eto + TBI)	9 (8.6)
Use of ATG	
No	62 (59.6)
Yes	42 (40.4)
Donor type	
Matched related	34 (32.7)
Mismatched related	0
Matched unrelated	35 (33.2)
Mismatched unrelated	13 (12.5)
Haploidentical donor	22 (21.6)
ABO matching degree	
Fully matched	50 (48.1)
Minor mismatched	18 (17.3)
Major mismatched	36 (34.6)
Stem cell source	
Peripheral blood	99 (95.2)
Bone marrow	5 (4.8)

*HCT-CI*, Hematopoietic Cell Transplant-specific Comorbidity Index; *RIC*, reduced intensity conditioning; *MAC*, myeloablative conditioning; *ATG*, anti-thymoglobulin; *Flu*, fludarabine; *Mel*, melphalan; *TBI*, total body irradiation; *Eto*, etoposide

### Hematological recovery and engraftment

All patients were evaluable for hematopoietic recovery and chimerism status. Patients received a median of 7.98 × 10^6^ CD34+ cells/kg (range 2.91 × 10^6^–16.98 × 10^6^ CD34+ cell/kg). After stem cell transplantation, engraftment was achieved at a median of 13.3 days for an ANC ≥ 500/μL and 15.2 days for platelet recovery (≥ 50,000/μL for 3 consecutive days without transfusion). All patients who underwent allo-HSCT had donor chimerism according to peripheral blood and showed chimerism data > 97% at 30 days post-transplant; full-donor chimeras were successfully completed in all patients.

### Survival outcomes after allo-HSCT

**Fig. 1:**
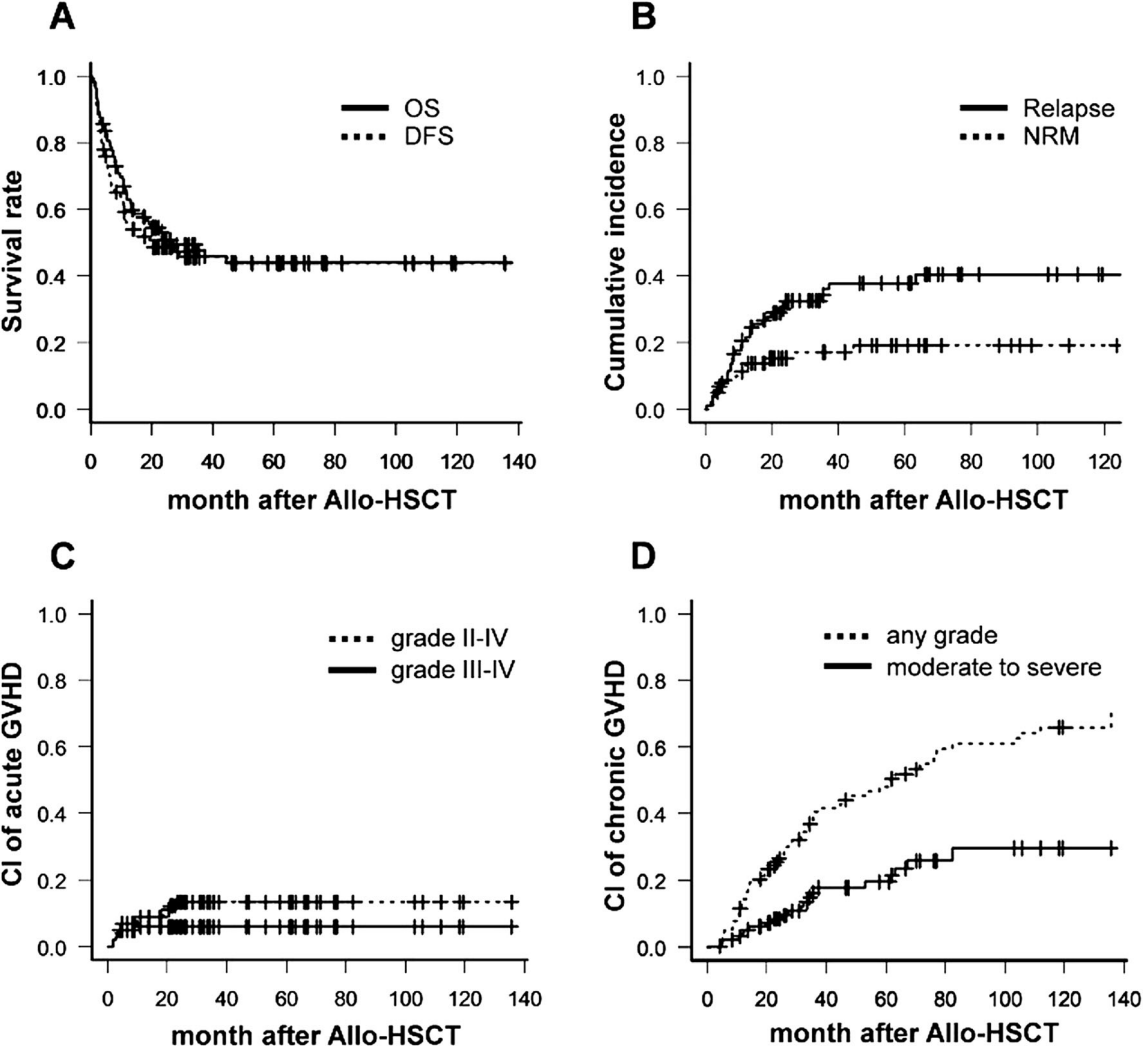
Survival outcome and GVHD incidence after allogeneic hematopoietic stem cell transplantation. **a** Overall survival and disease-free survival. **b** Cumulative incidence of relapse and non-relapse mortality. **c** Cumulative incidence of acute graft-versus-host disease (GVHD). **d** Represents chronic GVHD according to each severity

CR was observed in 68 patients (65.4%) after allo-HSCT. Among them, 23 patients (33.8%) had a pre-transplant disease status of refractory or relapsed to salvage chemotherapy or auto-HSCT. The median follow-up duration was 31.5 months (range 11.5–13.56 months) in surviving patients, and the median interval from the initial diagnosis to allo-HSCT in all patients was 19.8 months (range 5.6–86.2 months). The 1-year OS and DFS rates were 64.8% (95% confidence interval (CI) 54.7–73.2) and 64.7% (95% CI 54.7–73.0), respectively. The cumulative incidence rates of relapse and NRM at 1 year were 20.5% (95% CI 13.3–28.9) and 12.5% (95% CI 6.4–20.8), respectively (Fig. 1). The 3-year OS and DFS rates were 45.9% (95% CI 35.2–55.9) and 45.9% (95% CI 35.2–54.3), respectively. The cumulative incidence rates of relapse and NRM at 3 years were 36.0 (95% CI 26.1–46.0) and 17.0% (95% CI 9.5–26.5), respectively (Fig. 1).

### Graft-versus-host disease after allo-HSCT

**Fig. 2:**
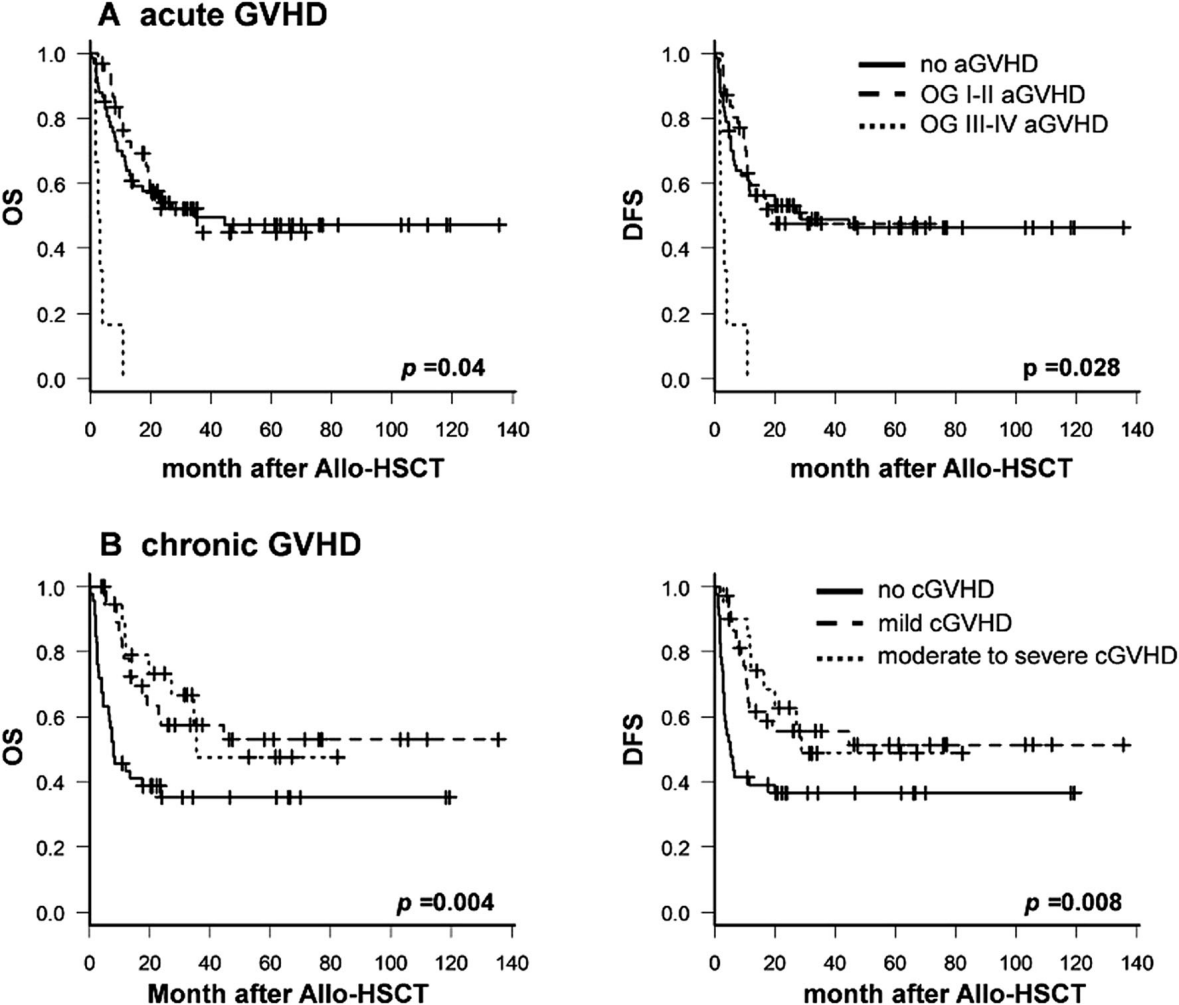
Survival outcomes according to acute GVHD and chronic GVHD. **a** Overall survival (OS) and disease-free survival (DFS) according to a grade of acute graft-versus-host disease (GVHD). **b** OS and DFS according to the severity of chronic GVHD

Any grade of acute GVHD occurred in 37 (35.6%) patients. Acute GVHD grades II–III was identified in 13 (12.5%) and 6 (5.8%) patients, respectively. Any stage of chronic GVHD was seen in 58 patients (55.8%). Furthermore, moderate and chronic stage GVHDs were identified in 14 (13.5%) and 6 (5.8%) patients, respectively (Fig. 1). Figure 2 panel a shows that patients with overall grades III–IV of acute GVHD had the markedly inferior OS and DFS than the group with overall grades I–II acute GVHD or no acute GVHD (*p* = 0.040 for OS and *p* = 0.028 for DFS, respectively). However, patients with more than mild stage chronic GVHD showed superior OS and DFS (*p* = 0.004 and 0.008, respectively) (Fig. 2 panel b). Furthermore, more than moderate stage chronic GVHD was still superior in terms of OS compared with non-chronic GVHD, and DFS did not differ in patients with non-chronic GVHD (Fig. 2 panel b).

### Clinical factors associated with GRFS

**Fig. 3:**
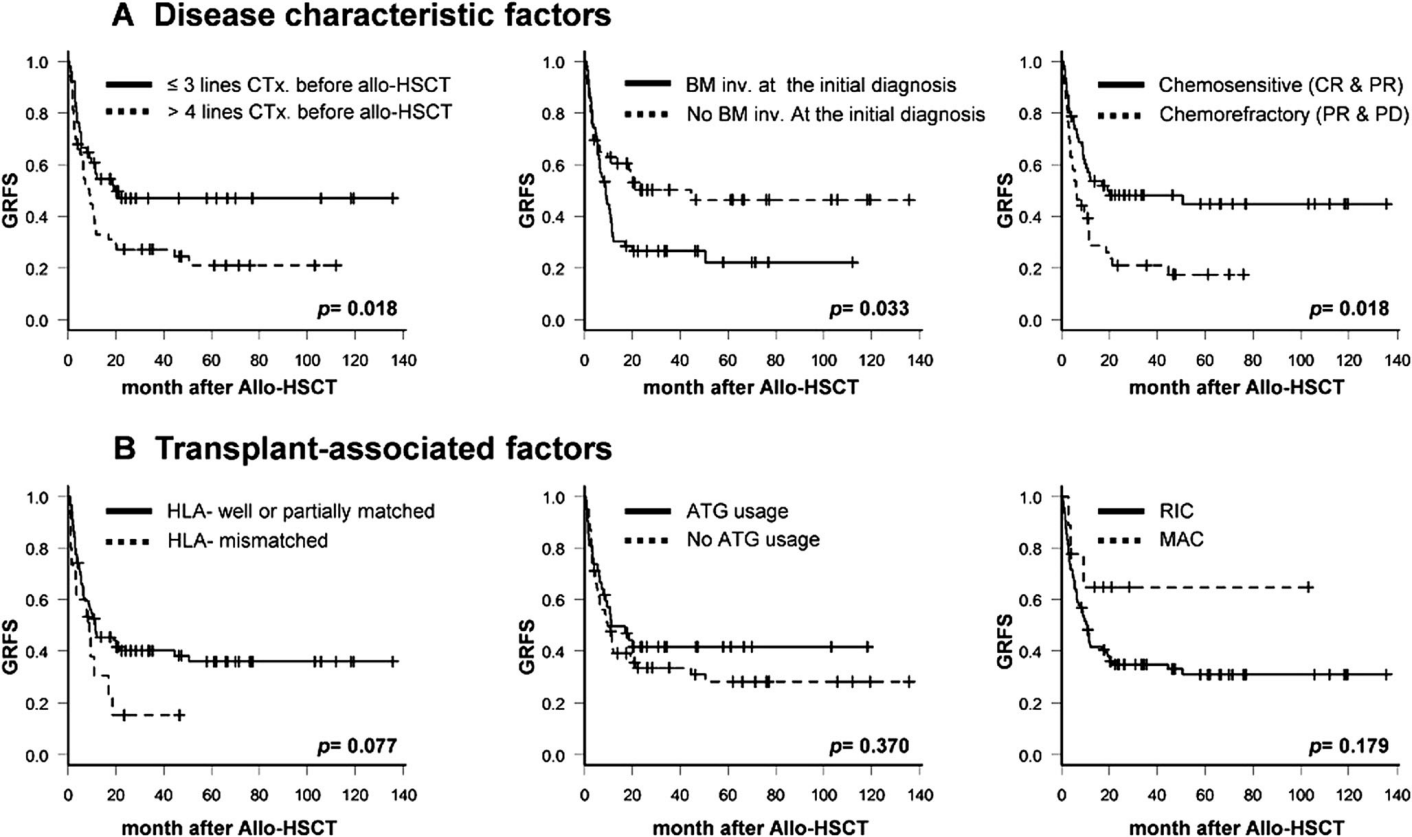
Clinical factors influencing GVHD-free with relapse-free survival (GRFS). **a** Disease characteristic factors associated with GRFS; GRFS differed according to cumulative lines of chemotherapy before allo-HSCT, bone marrow involvement at diagnosis, and disease status prior to allo-HSCT. **b** Shows that transplant-associated factors influencing GRFS; GFRS differed according to HLA matching degree, usage of anti-thymocyte globulin, and conditioning intensity

The GRFS rate at 1 year after allo-HSCT was 44.5% (95% CI 34.7–53.8) in the entire cohort, and all comparisons with OS (64.8%; 95% CI 54.7–73.2) and DFS (64.7%; 95% CI 54.7–73.0) during the same period are shown in Supplementary Figure 1. Also, the 3-year GRFS rate was 36.9% (95% CI 27.5–46.3) compared with an OS of 45.9% (95% CI 35.2–55.9) and DFS of 45.9% (95% CI 35.2–54.3) (Supplementary Figure 1). The comparative analysis of GRFS and clinical factors was performed by classifying the factors related to the disease characteristics and the factors related to the transplant features. In the analysis of lymphoma-specific characteristics factors, 3-year GFRS was significantly favorable in patients with ≤ 3 lines of chemotherapy before allo-HSCT (47% vs. 27%, *p* = 0.018), no BM involvement at the initial diagnosis (50% vs. 27%, *p* = 0.033), and chemosensitive disease status before transplant (48% vs. 21%, *p* = 0.018) (Fig. 3 panel a). However, there were no major modifiable transplant-associated factors which correlated with the 3-year GRFS incidence, including of HLA matching degree (40% vs. 15% in HLA well or partially matched vs. mismatched, *p* = 0.077), use of ATG (42% vs. 34% in with ATG vs. without ATG, *p* = 0.370), conditioning intensity (65% vs. 35% in MAC vs. RIC, *p* = 0.179), the Hematopoietic Cell Transplant-specific Comorbidity Index (HCT-CI), donor type (sibling or unrelated), and stem cell source (BM or peripheral blood) (Fig. 3 panel b).

### Causes of GRFS events

**Fig. 4:**
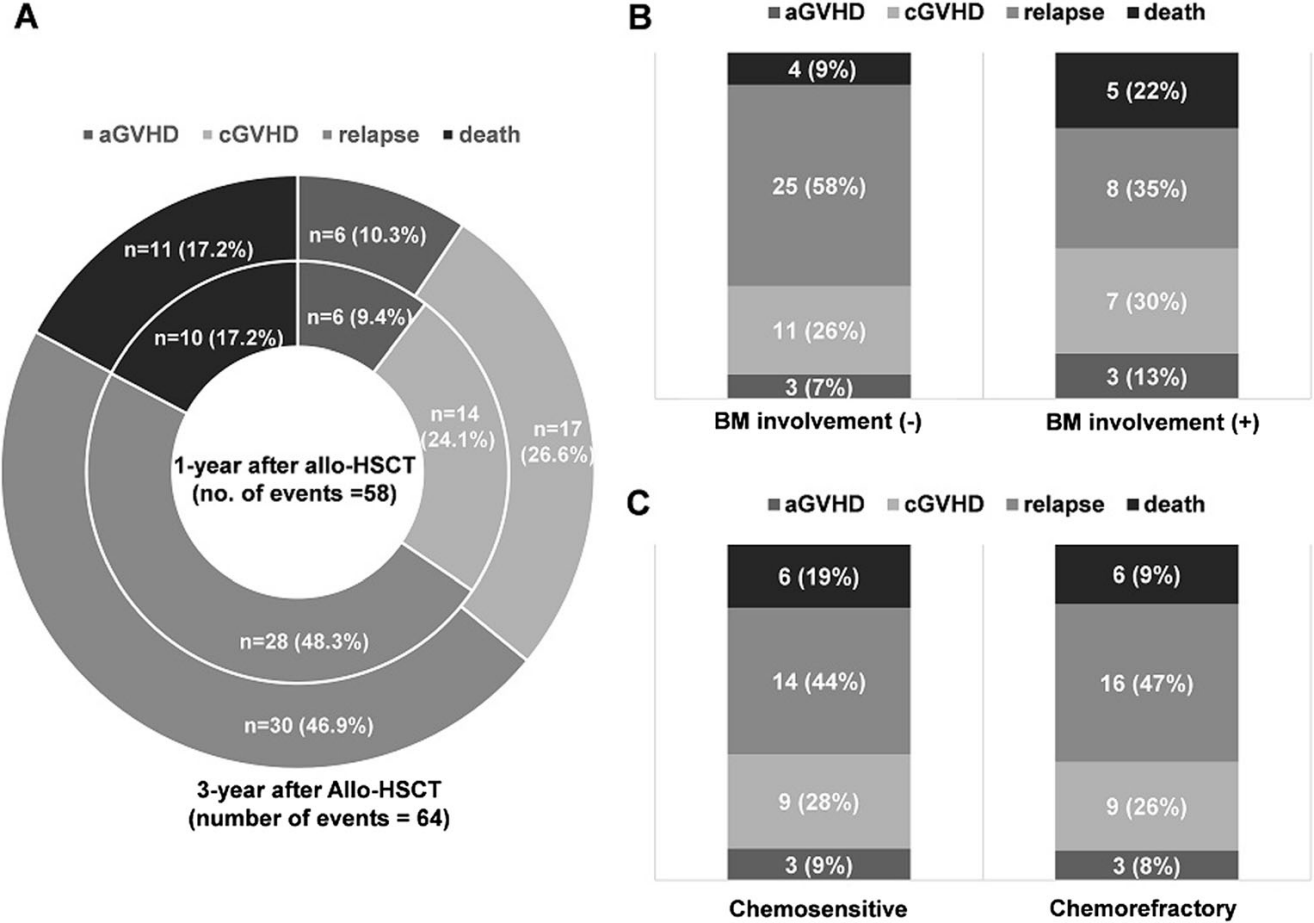
Distribution of GRFS-defining events after allo-HSCT in patients with refractory or relapsed NHL. Acute GVHD, chronic GVHD, relapse rate, and death rate differed between 1-year and 3-year after allo-HSCT (A). BM involvement (**b**) and disease status pre-HSCT (**c**) impact GRFS incidence

To investigate whether the distribution of GRFS-defining events was due to the incidence of relapse or the GVHD incidence and NRM rate, we analyzed the impact of each of these events on cumulative incidence. Among the entire 104 patient cohorts, 58 patients experienced a GRFS event within 1 year after allo-HSCT, and 64 patients had involved a GRFS event within 3 years after transplant. Relapse (*n* = 28, 48.3%) accounted for the most significant proportion of GRFS events in the definitive 1-year GRFS, and the frequency of GRFS-related events was high in the order of chronic GVHD, death, and acute GVHD (24.1%, 17.2%, and 9.4% respectively). Similar to 1-year GRFS, the events associated with the crude 3-year GRFS were more frequent in the order of relapse, chronic GVHD, death, and acute GVHD (46.9%, 26.6%, 17.2%, and 10.3% respectively) (Fig. 4a). Besides, each of the individual GRFS-related event analysis was performed for known independent factors affecting GRFS, such as BM involvement or disease status during the pre-HSCT period. Both factors showed frequency results in the order of relapse, chronic GVHD, death, and acute GVHD.

## Discussion

We analyzed 104 consecutive adult patients with refractory or relapsed aggressive NHL alone receiving allo-HSCT, using the novel concept of GRFS to avoid possible confounding factors due to various clinical presentations and therapeutic approaches for other hematological malignancies. We also further explored the impact of each clinical parameter on GRFS in patients with NHL. Using GRFS as an endpoint, we found that 44.5% and 36.9% of patients with advanced NHL survived for 1 and 3 years, respectively, after allo-HSCT without any GRFS-related events. Survival outcomes, including GRFS, reached a plateau within 3 years after allo-HSCT. The median time to present with a GRFS-related event was 11 months after allo-HSCT, and the majority occurred from 1 month to 3 years. Although it is difficult to directly compare GRFS with other hematological malignancies due to variation in disease-specific characteristics and HSCT approach, our study showed a favorable GRFS rate compared with results previously reported for other hematological malignancies such as acute leukemia or myelodysplastic syndrome [10, 18, 19], and a similar GRFS rate compared with the previous rate of 30–45% GRFS at 1–3 years in patients with lymphoma [20, 21].

Dodero et al. [20] reported favorable outcomes (61% and 50% 3-year OS and PFS, respectively) with reasonable GRFS (34% at 3 years) in a relatively homogenous group by treating with the RIC regimen and adding rituximab for GVHD prophylaxis. However, our entire cohort showed similar survival outcomes and GRFS incidence, despite more unfavorable transplant-circumstances such as SD/PD status before HSCT or unrelated/haploidentical donors.

When the factors related to an increment in GFRS incidence were analyzed, event incidence of GRFS was higher in patients with more cumulated lines of chemotherapy before allo-HSCT, an involved BM at initial diagnosis or chemorefractory disease status before transplant. However, these were merely fixed biological factors that were determined by initial disease status, and no transplant-associated variables were found. In various hematology malignancies, Holtan et al. [10] suggested that adjust clinical factors, such as BM source or donor type, among the transplant-associated factors. However, our cohort had no adjustable transplant-related factors including donor type, donor age, conditioning intensity, HLA matching degree, and HCT-CI. To explain these results, we investigated the incidence of each GRFS-defining event. Figure 4 shows that relapse was the most common event type among GRFS-related events at 1 and 3 years after allo-HSCT (Fig. 4a). A relapse event was still the most frequent type after performing individual analyses according to BM involvement or pre-HSCT chemotherapy response status (Fig. 4b, c). In other words, because patients with NHL are inevitably exposed to many chemotherapeutic drugs before allo-HSCT is induced, disease status at the pre-transplant period eventually dominates the final survival outcomes after allo-HSCT. Thus, it means that the most crucial factor to improve the GRFS rate of NHL patients is a controlled disease state before allo-HSCT.

According to the previously reported GRFS studies for lymphoma only, the poor GRFS rate was associated with an aggressive pathologic subtype, a prolonged BM involvement, and a related donor stem cell source in B cell lymphoma [20]. Another research for GRFS rate by Gauthier et al. [21] reported similar results in Hodgkin’s lymphoma. These studies also identified that haploidentical donors who received ATG had good GRFS compared with mismatched unrelated donors. Based on these results, we also investigated whether the incidence of GRFS was increased by recipients of BM grafts from haploidentical donors (*n* = 22, 21.6%), MAC regimen (*n* = 9, 8.6%), pathologically aggressive NHL (*n* = 37, 35.6%), and ATG usage (*n* = 42, 40.4%). However, our results showed that GRFS incidence was not different from these clinical factors. The incidence of chronic GVHD differs by study, and we used the same RIC regimen for both haploidentical donors and related/unrelated donors; the cumulative incidence of chronic GVHD was relatively high in our center (approximately 10% vs. 22% of cumulative incidence of chronic GVHD), and it was expected that this diversity would impact on GRFS.

**Fig. 5:**
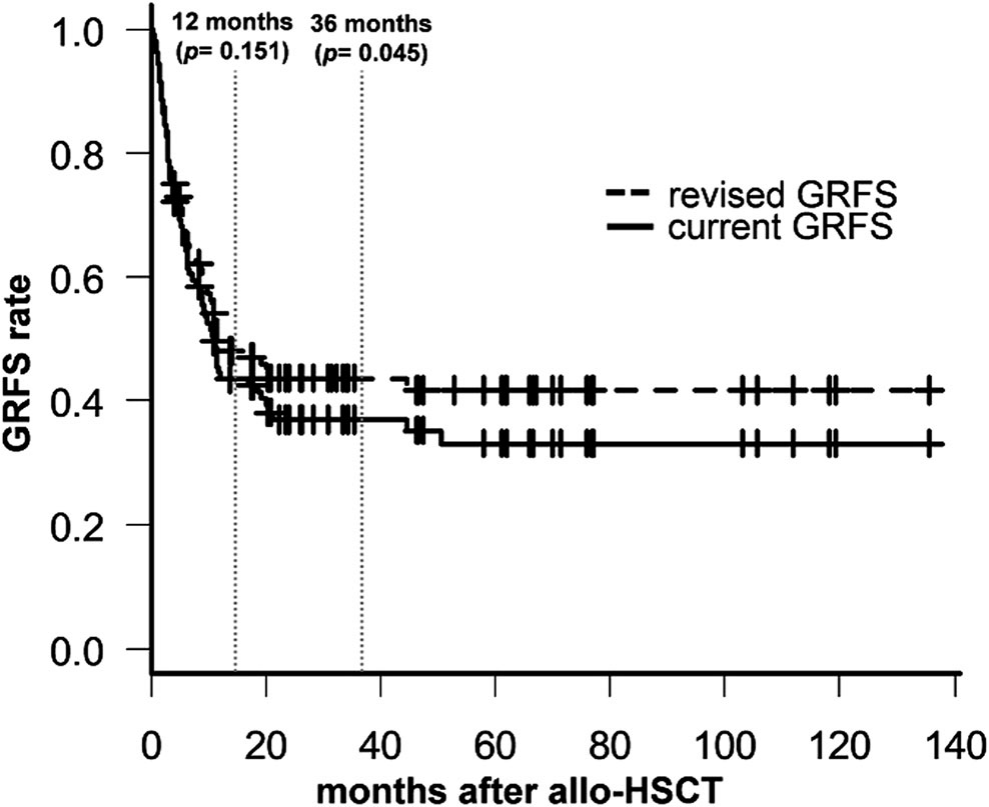
Comparison of conventional and revised GRFS. The analysis was performed using the revised dynamic GRFS method, except for fully restored chronic GVHD events for short-term systemic immunosuppressive therapy. The results show the advantage of survival compared with the GRFS method

Then, one way to improve GRFS in patients with refractory or relapsed NHL is to control GVHD events, as a relapse event is not considered by the transplant approach only. We preferentially analyzed the association between the incidence of acute/chronic GVHD and survival outcomes. The cumulative incidence rates of grades III–IV acute GVHD and systemic therapy-requiring chronic GVHD at 3 years were 5.8% and 17.6%, respectively (Fig. 1). Acute GVHD did not affect OS or cumulative incidence of relapse (CIR) rates and did not increase NRM further (*p* = 0.478, 0.449, and 0.754, respectively; Supplementary Figure 2-A, C, and E). In contrast, chronic GVHD improved OS and lowered the CIR rate, but did not increase NRM (*p* < 0.001, 0.021, and 0.858, respectively; Supplementary Figure 2-B, D, and F). A possible explanation for this finding is the GVL response; several preclinical and retrospective clinical studies have reported a similar GVL effect in lymphoma [20, 22–24] and in chronic GVHD, but not in acute GVHD, further increasing the GVT effect [25]. Moreover, the low-dose TBI-based nonmyeloablative conditioning regimen relies nearly exclusively on the intensified GVL effect [26, 27]. Because the method used to calculate GRFS considers chronic GVHD as a fixed negative event, such as relapse or death, and even though chronic GVHD may be fully resolved by short-term treatment, this GRFS method tends to overestimate the impact of chronic GVHD on the outcome of allo-HSCT, particularly in patients with NHL. Solomon et al. [28] and Kawamura et al. [19] presented a dynamic GRFS rate in which GVHD was resolved by short-term treatment in patients with acute leukemia. Therefore, we recalculated the current GRFS, except in patients whose chronic GVHD was fully resolved over a less than 1-month course of systemic immunosuppressants, and analyzed these patients using the revised dynamic GRFS method. As shown in Fig. 5, survival increased over time (36.9% vs. 41.9%, *p* = 0.045 for current GRFS vs. revised GRFS at 3 years after allo-HSCT). Thus, we confirmed that chronic GVHD was necessary to achieve the GVL effect in our cohort, and that rapidly controllable chronic GVHD was considered without continuing morbidity or decreased QoL by the ongoing moderate to severe chronic GVHD.

Another way to enhance GRFS by reducing GVHD events is to utilize T cell-replete grafts, which administrated with intensive immunosuppression using ATG or infusing PT-CY [29]. In this study, we used only ATG as T cell-replete method, which did not affect the incidence of GRFS. This is considered due to heterogeneous subtype and a small number of patients using ATG. However, Kanate et al. [30] reported very promising data related to PT-CY method in haploidentical transplantation for lymphoma: T cell-replete strategy with PT-CY showed that relapse risk, NRM, DFS, and OS were similar with non-PT-CY. However, a risk of grades III–IV acute and chronic GVHD was significantly lower with haploidentical transplantation of PT-CY compared with others. Based on these results, although there were no patients with allo-HSCT utilizing PT-CY method in our study, it is strongly possible to predict that GRFS might be improved if using selective PT-CY strategy in haploidentical transplantation.

In conclusion, our results indicate that allo-HSCT for patients with refractory or relapsed NHL alone showed a favorable GRFS rate compared with other previously reported studies for several hematological malignancies, despite the complex pathologic subtypes and variable therapeutic courses of the diseases. We did not identify any modifiable clinical transplant-associated factors that were previously reported in several acute leukemia cohorts. However, BM involvement, pre-HSCT disease status, and exposure lines of chemotherapy before transplantation were related to the GRFS rate, and these results were assumed to be due to the relapse events that were the major consideration in the analysis of GRFS-related factors. GVHD was shown to be the next most important factor to improve GRFS in patients with relapsed or refractory NHL; it was confirmed that the GVL effect positively influenced survival outcomes, and rapidly resolved chronic GVHD did not result in continued morbidity/mortality or decreased QoL due to the revised dynamic GRFS. Therefore, considering (and controlling) chronic GVHD as a dynamic event rather than a static one may improve diagnostic accuracy; it is reasonable to assume that resolving chronic GVHD during short-term treatment is a practical way for measuring GRFS in patients with refractory or relapsed NHL only.

## Electronic supplementary material

ESM 1 (PDF 126 kb)
