# Supplementary material for: Risk factors predicting graft-versus-host disease and relapse-free survival after allogeneic hematopoietic stem cell transplantation in relapsed or refractory non-Hodgkin’s lymphoma
Source: Ann Hematol. 2019 May 14;98(7):1743–53. doi: 10.1007/s00277-019-03714-x (PMC6591200; doi:10.1007/s00277-019-03714-x)

Supplementary Figure 1. Comparison of overall survival (OS), disease-free survival (DFS), and GRFS of all patients

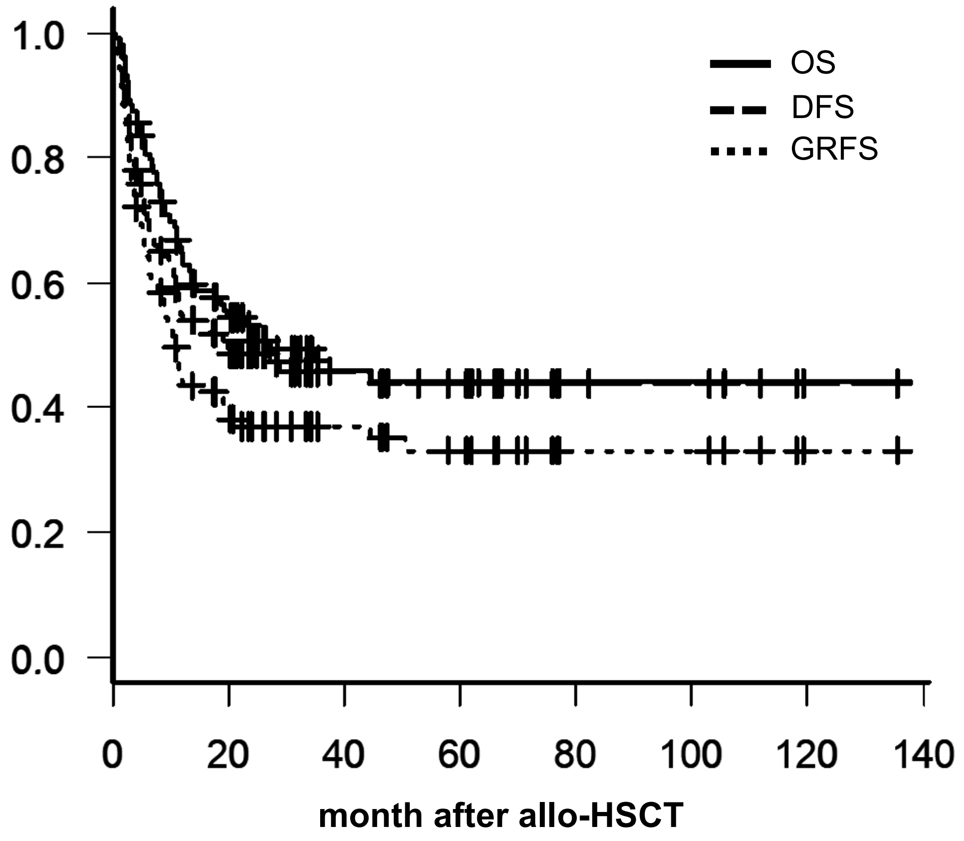

Supplementary Figure 2. Survival outcomes according to GVHD

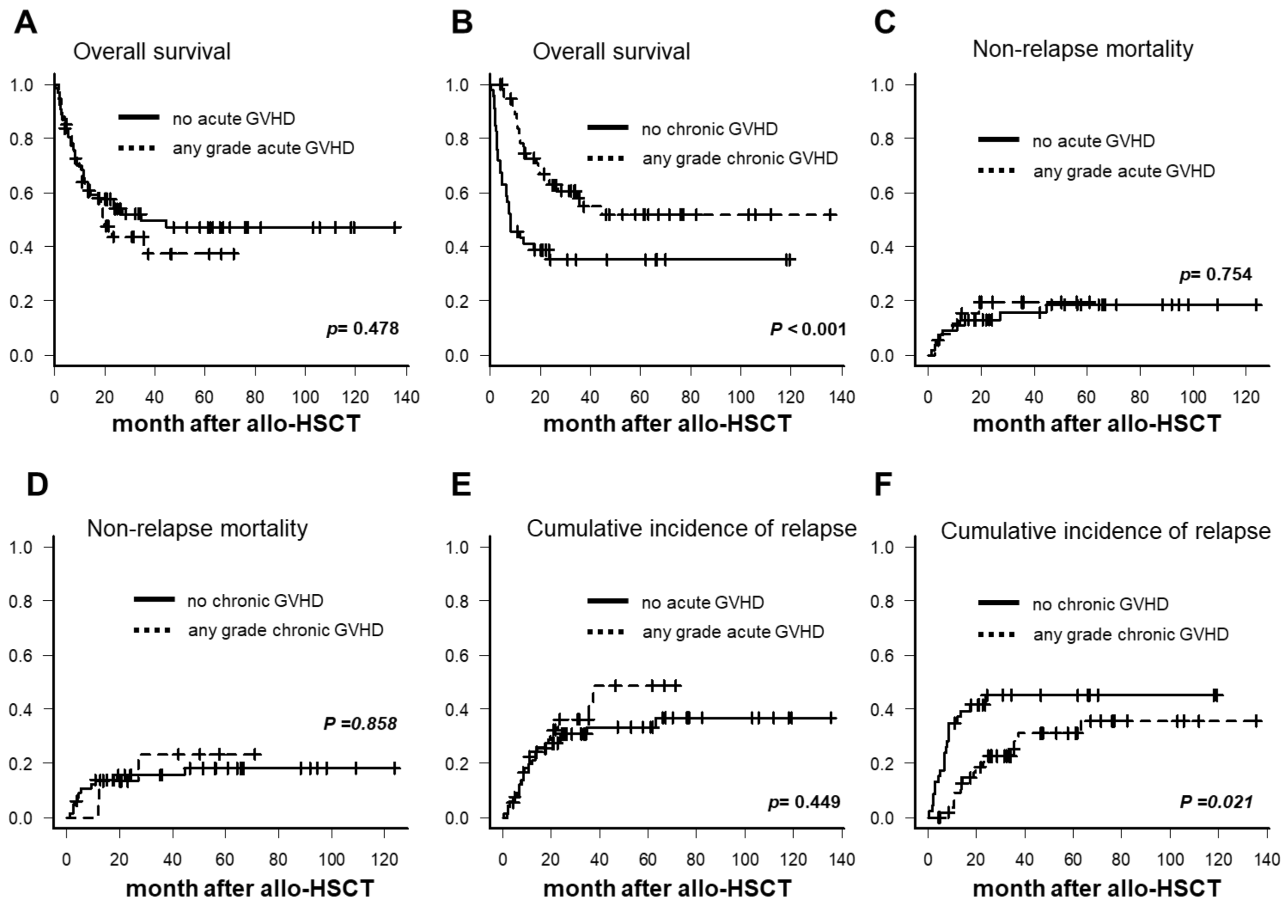

Supplement: Supplementary file 1 — (PDF 126 kb) [file 277_2019_3714_MOESM1_ESM.pdf]
